# Supplementary material for: 16S rRNA sequencing reveals relationships among enrichment of oral microbiota in the lower respiratory tract and pulmonary nodules malignant progression
Source: Microbiol Spectr. 2025 Feb 5;13(3):e01284-24. doi: 10.1128/spectrum.01284-24 (PMC11878090; doi:10.1128/spectrum.01284-24)
Supplement: Figure S1 — Oral microbiome differences in alpha diversity for chao1 (A), Sobs (B), pd (C), Shannon (D), and Simpson (E) index. [file spectrum.01284-24-s0001.pdf]

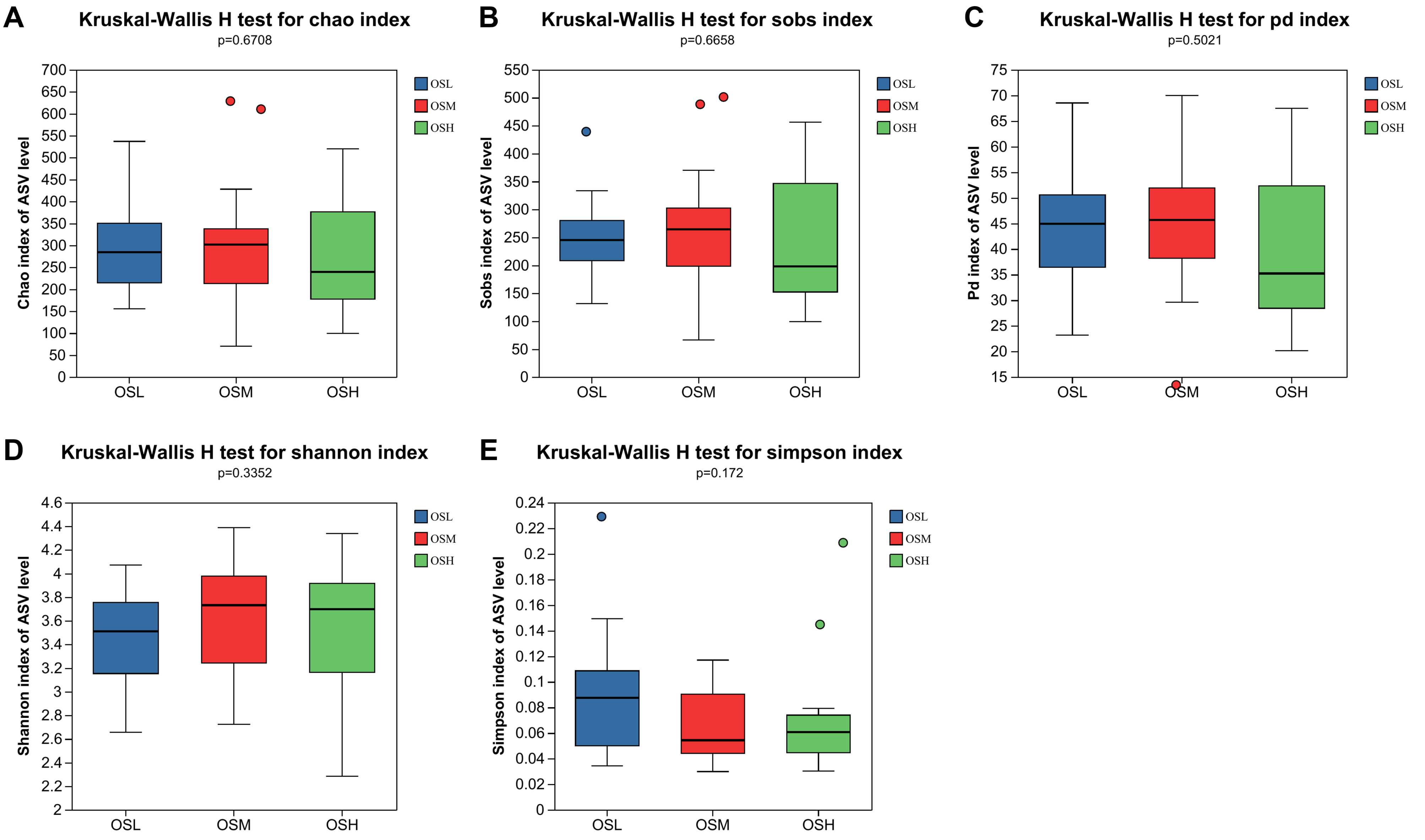

Fig.S1 Oral microbiome differences in alpha diversity for chao1 (A), Sobs (B), pd (C), Shannon (D), and Simpson (E) index.
