# Supplementary material for: 16S rRNA sequencing reveals relationships among enrichment of oral microbiota in the lower respiratory tract and pulmonary nodules malignant progression
Source: Microbiol Spectr. 2025 Feb 5;13(3):e01284-24. doi: 10.1128/spectrum.01284-24 (PMC11878090; doi:10.1128/spectrum.01284-24)
Supplement: Figure S2 — The comparative analysis of differential bacterial genera in oral cavity. [file spectrum.01284-24-s0002.pdf]

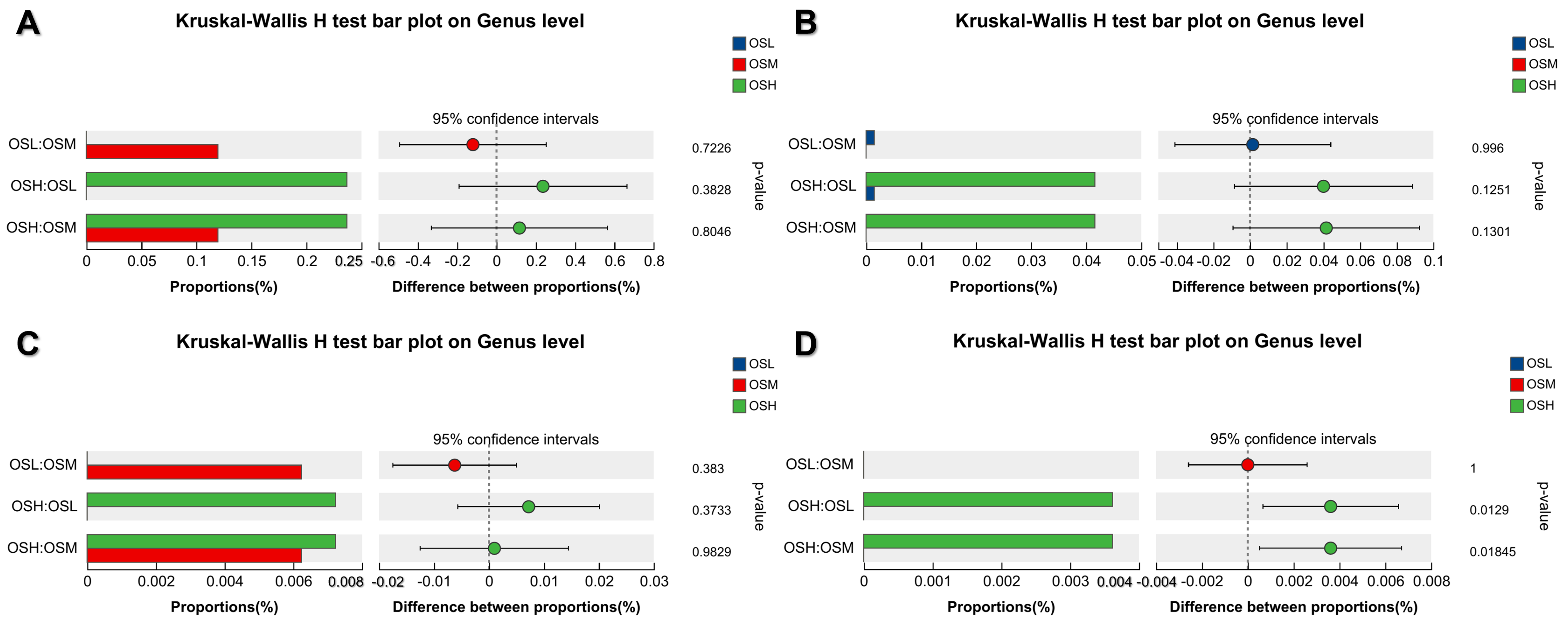


Fig.S2 The comparative analysis of differential bacterial genera in oral cavity. (A) Undibacterium; (B) Staphylococcus; (C) Pseudoramibacter; (D) Synergistes.
