# Supplementary material for: 16S rRNA sequencing reveals relationships among enrichment of oral microbiota in the lower respiratory tract and pulmonary nodules malignant progression
Source: Microbiol Spectr. 2025 Feb 5;13(3):e01284-24. doi: 10.1128/spectrum.01284-24 (PMC11878090; doi:10.1128/spectrum.01284-24)
Supplement: Figure S3 — The comparative analysis of differential bacterial genera in LRT. [file spectrum.01284-24-s0003.pdf]

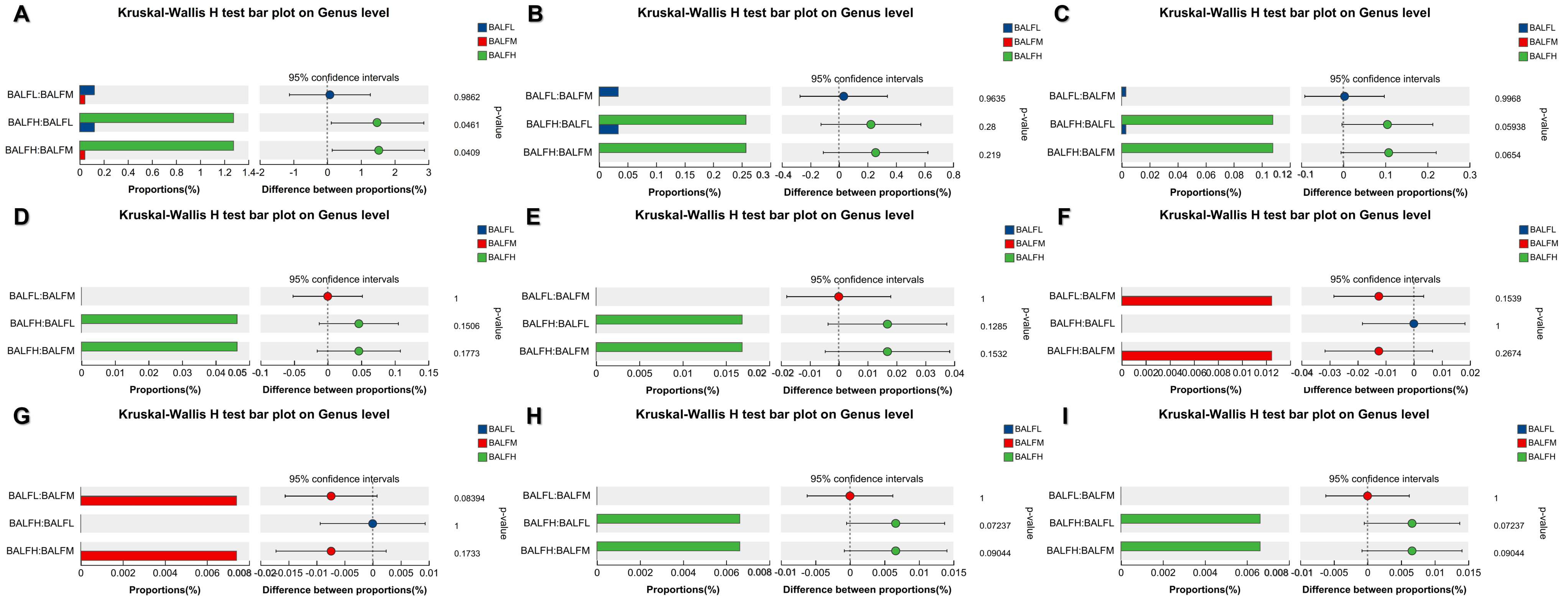

Fig.S3 The comparative analysis of differential bacterial genera in LRT. (A) Tannerella; (B) F0058; (C) Carnobacterium; (D) Synergistes; (E) UCG-005; (F) Roseomonas; (G) Lachnoclostridium; (H) Odoribacter; (I) Mycobacterium.
