# Supplementary material for: 16S rRNA sequencing reveals relationships among enrichment of oral microbiota in the lower respiratory tract and pulmonary nodules malignant progression
Source: Microbiol Spectr. 2025 Feb 5;13(3):e01284-24. doi: 10.1128/spectrum.01284-24 (PMC11878090; doi:10.1128/spectrum.01284-24)
Supplement: Figure S4 — The association between oral microbiota and LRT microbiota in low-risk PN patients [file spectrum.01284-24-s0004.pdf]

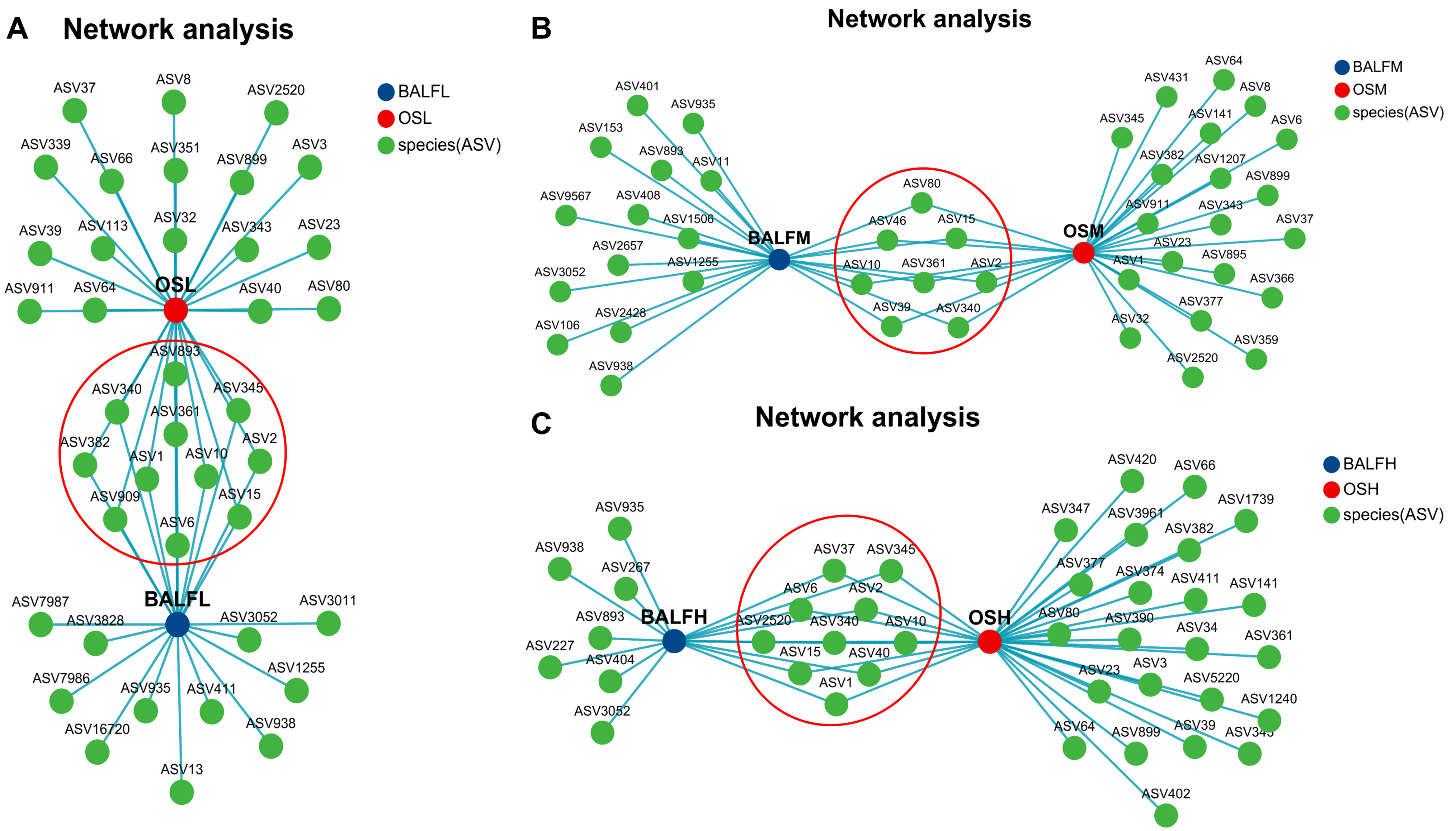

Fig.S4 The association between oral microbiota and LRT microbiota in low-risk PN patients (A); medium-risk PN patients (B); high-risk PN patients (C). Each node represented an ASV, and the circles indicate the shared dense clusters in both groups.
