# Supplementary material for: 16S rRNA sequencing reveals relationships among enrichment of oral microbiota in the lower respiratory tract and pulmonary nodules malignant progression
Source: Microbiol Spectr. 2025 Feb 5;13(3):e01284-24. doi: 10.1128/spectrum.01284-24 (PMC11878090; doi:10.1128/spectrum.01284-24)
Supplement: Figure S5 — ROC analysis of (A) oral differential bacteria genus Synergistes; (B) LRT differential bacteria genus Tannerella; (C) Rothia</>; (D) Veillonella. [file spectrum.01284-24-s0005.pdf]

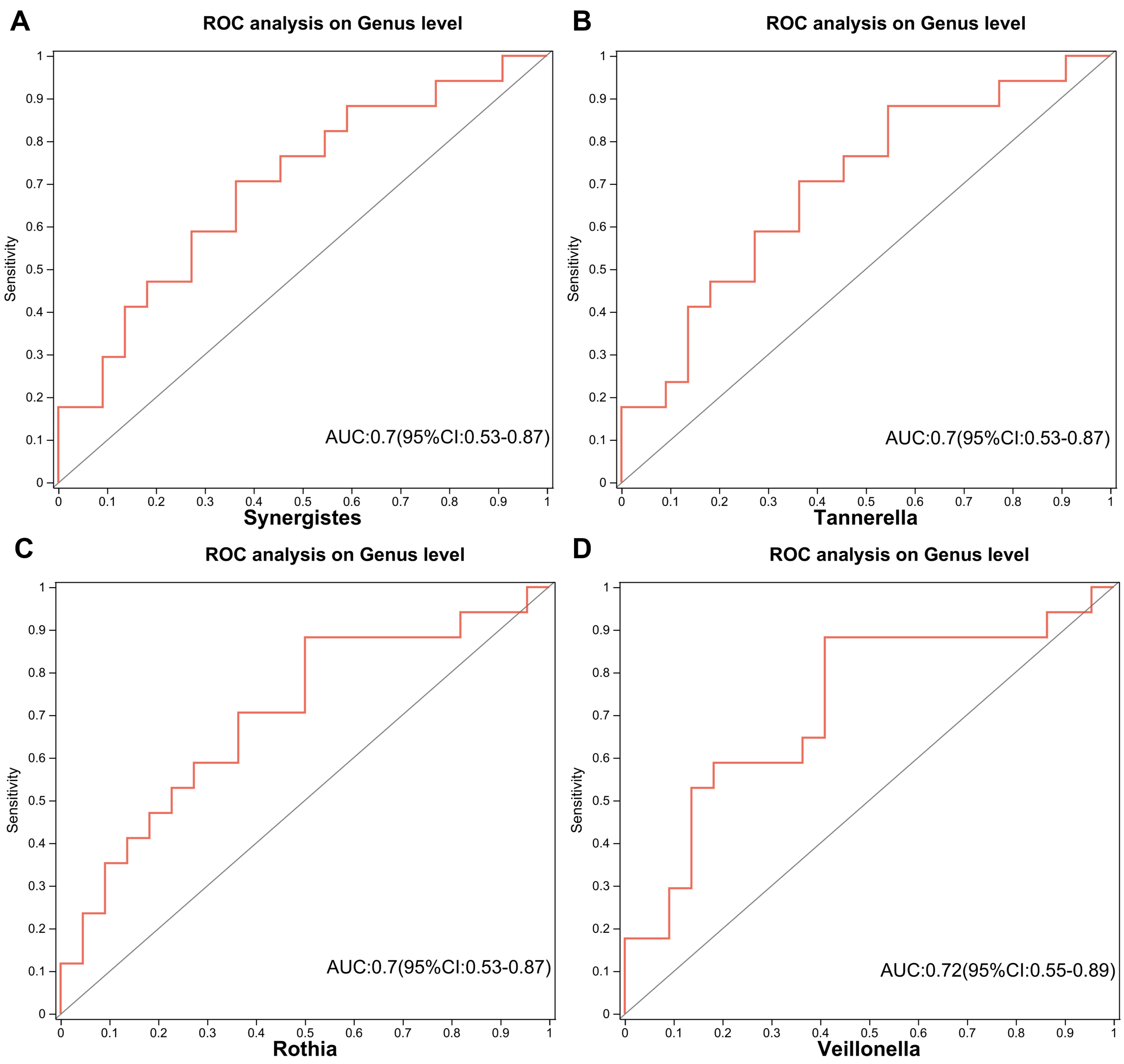

Fig.S5 ROC analysis of (A) Oral differential bacteria genus Synergistes; (B) LRT differential bacteria genus Tannerella; (C) Rothia; (D) Veillonella.
